# Violence as an individual concern: responding to technology-facilitated sexual violence within child and adolescent psychiatry

**DOI:** 10.3389/fpsyg.2025.1602655

**Published:** 2025-06-27

**Authors:** Frida Carlberg Rindestig, Katja Gillander Gådin, Linda Jonsson, Inga Dennhag

**Affiliations:** ^1^Child- and Adolescent Psychiatry, Department of Clinical Science, Affiliated With the Graduate School of Gender Studies, Umeå University, Umeå, Sweden; ^2^Department of Health Sciences, Mid Sweden University, Sundsvall, Sweden; ^3^Department of Social Sciences, Marie Cederschiöld University, Stockholm., Sweden; ^4^Child- and Adolescent Psychiatry, Department of Clinical Science, Umeå University, Umeå, Sweden

**Keywords:** technology facilitated sexual violence, child and adolescent psychiatry, psychiatrization of society, reflexive thematic analysis, interviews - methods, young people

## Abstract

Technology-facilitated sexual violence (TFSV) is an emerging concern in child and adolescent mental health. This article presents an analysis of how child and adolescent psychiatric professionals conceptualize the phenomenon of TFSV in relation to their clinical practice with patients exposed to this type of violence. Through Reflexive Thematic Analysis, we constructed the overarching theme: *Violence as an Individual Concern.* Our findings are analyzed through the lens of psychiatrization—a societal process that extends the influence of psychiatry, making social issues into psychiatric problems. This research provides critical insights into how the medicalization of TFSV may inadvertently individualize this broader societal issue.

## Introduction

The rise of digital technology has transformed how interpersonal violence is perpetrated, introducing new arenas for abuse that need to be considered when addressing violence in society. Technology-facilitated sexual violence (TFSV) is one such phenomenon. Research into TFSV is growing, delving into the causes and consequences of being exposed to sexual violence with the aid of technology. However, there is a lack of investigation into how relevant societal actors, such as Child and Adolescent Psychiatry (CAP), respond to this phenomenon in their institutional practices. This article examines how CAP professionals in Sweden conceptualize and address TFSV, offering insights into psychiatric institutional responses to this complex phenomenon.

The study will begin by providing a brief overview of research relevant to the phenomenon of TFSV, setting the context for how it has been studied.

Regarding the definition of TFSV, scholarly literature has, in general, kept the definition broad, in line with definitions of In Real Life (IRL) sexual violence. The definition used in this article draws on Henry and Powell, which defines TFSV as “a range of criminal, civil, or otherwise harmful sexually aggressive and harassing behaviors that are perpetrated with the aid or use of communication technologies” (Henry and Powell, 2018).

The prevalence of TFSV in general populations varies depending on the specific type of TFSV, as well as on how the phenomenon is operationalized and measured. Online sexual solicitation has generally been shown to be the most common, while webcam livestreamed abuse is relatively rare (Finkelhor et al., 2024; Madigan et al., 2018).

Prior research has identified significant associations between TFSV and mental health difficulties, underscoring the profound impact of such violence. Regarding mental health, TFSV is associated with mental health complaints in much the same extent as IRL sexual violence (e.g., Joleby et al., 2020a; Joleby et al., 2020b; Jonsson et al., 2019) and is shown to have long-term negative social and mental health consequences for children when they enter adulthood (Schmidt et al., 2023b; Schmidt et al., 2023a). These associations should be interpreted in light of broader structural and contextual factors. When focusing on the population in CAP, factors known to be essential for mental health are routinely considered in assessments of children and young people. One crucial such factor is interpersonal violence, known to be highly associated with mental health. This is shown to be particularly common in the CAP population (e.g., Ford et al., 2011; Hultmann and Broberg, 2016; Hultmann et al., 2022), making different forms of violence a target for assessment in clinical practice. Further, different forms of violence exposure tend to cluster together, making comprehensive screening essential to account for the whole burden of violence among young people in CAP-care (Wolfe, 2018). Given the expansion of interpersonal violence into online arenas, it is imperative to also include technology-related violence in such assessments, particularly sexual subtypes, since sexual violence is known for its profound negative effects on mental health (Chen et al., 2010; Dworkin, 2020; Rajan et al., 2020).

Which institutions should be responsible for responding to the issue of TFSV are not clearly outlined, but legal authorities, social services, as well as mental health care are actors that could become involved in both prevention and addressing its aftermath. How the institutions formulate their task in this matter remains understudied.

Existing research in the area has primarily investigated how professionals feel and think about the phenomenon, as well as how they clinically respond to patients with experience of different forms of TFSV. These studies have a realist framework and highlight how professionals in mental health care perceive and address TFSV in their clinical practice. The findings suggest that many professionals in mental health care are lagging in knowledge about the new arenas and modes of perpetration that online technology has introduced, and are often not taking TFSV as seriously as acts perpetrated IRL (Binford, 2023; Dimitropoulos et al., 2022; Hamilton-Giachritsis et al., 2021; Quayle et al., 2023; Schmidt et al., 2023b; Schmidt et al., 2023a). Furthermore, professionals often describe TFSV as a complex and multifaceted phenomenon, finding it difficult to grasp. They also perceive specific organizational barriers addressing the problem adequately, such as a lack of explicit questions or screening procedures to identify these patients (Quayle et al., 2023). A study of mental health organizations targeting young people reveals that few have specific strategies in place for working with TFSV (Schmidt et al., 2023b; Schmidt et al., 2023a). One of the most salient issues that frontline professionals describe that they come across when meeting young people exposed to different types of TFSV, is how to address the permanence of the abuse images living their own life online (Binford, 2023). The interpretations and conclusions of these studies are framed in realist terms, where knowledge gaps about the phenomenon of TFSV, such as a lack of knowledge regarding epidemiology or mental health consequences, are emphasized. They also conclude that there is a need for evidence-based therapies or approaches to address this specific phenomenon among children and young people.

From another perspective, understanding an institution’s approach to addressing an issue can be achieved by studying how it frames and formulates the problem in question. The way they frame an issue can be explicitly found in policies around how to address the issue, as well as in less formalized ways reflected in professional culture, or the issue is discussed within the organization or professional group.

To the authors’ knowledge, no studies have investigated how professionals in mental health care, viewed as representatives of larger institutional discourses or narratives, frame the problem of TFSV. This is an essential expansion of the growing body of knowledge about professionals’ experiences of working with TFSV, adding a focus on a more discursive level that can help to address gaps in understanding within organizations working with mental health in young people. This can ultimately help organizations to address the problem more comprehensively. The present study, therefore, explores how CAP professionals in Sweden conceptualize and address TFSV, providing insights into institutional conceptualizations of this complex phenomenon. We had a broad definition of TFSV, including all types of sexual abuse or exploitation via online communication technologies.

### The research question

The research question guiding the analysis in the present study was “How do professionals working in CAP in Sweden conceptualize the phenomenon of TFSV about their work and their young patients?”

## Materials and methods

This particular study is a part of a larger project aimed at charting the mental health service needs of victims of TFSV and the present supply of services that child and adolescent psychiatry CAP, as well as social services and the police, provide to victims of TFSV. The interview guide for CAP professionals focused on how participants, as mental health care providers within CAP, perceived victims of TFSV, how they conceptualized their problems, how they responded to patients’ needs for mental health care, and how they collaborated with other institutions.

### Participants

The participants (*N* = 12) were professionals working in CAP clinics in Sweden. An additional sample (*N* = 7) of former CAP professionals who had advanced to higher level employments in the field, such as executives of child advocacy centers or those holding government commissions, was also included. They are part of the knowledge production in the field, which, in turn, governs CAP as an organization and outlines how they should work with these patients. Inclusion criteria were being a (current or former) healthcare professional in a CAP clinic in Sweden, as well as having at least some experience of meeting young people being sexually abused through the aid of online technology. We had a broad definition of TFSV, including all types of sexual abuse or exploitation via online communication technologies. All professionals had met some, but most of them several or many, patients where TFSV had been a problem. The types of TFSV that the professionals had encountered ranged from pressure to send nude images, images used as leverage for sexual extortion, grooming leading to both online and offline sexual abuse, as well as online sexual abuse via webcam.

The professional backgrounds of the participants varied, but the vast majority were psychologists, followed by psychotherapists and social workers. The sample consisted of all females. This is largely reflective of the gender distribution of most CAP clinics in Sweden, with women constituting the majority of the staff.

### Design

We employed an interview-based design with qualitative data material in the form of texts derived from transcribed interviews with professionals working in CAP services. The interviews were held by several different interviewers employed within the project. A snowball sampling strategy was used to recruit participants. Snowball sampling was used, as CAP professionals rarely have the time to respond to impersonal advertisements, making directed contacts more likely to result in participation.

The interviews were semi-structured, following a set of questions or topics, but with room for follow-up questions and clarifications as needed. Additionally, the questions were formulated in a way that made the interview less interrogative and more conversational.

Approximately half of the interviews were transcribed with the aid of Whisper software, supported by the Swedish national research infrastructure, Språkbanken and Swe-Clarin. The transcripts were then double-checked to ensure they matched the audio files and edited as necessary. The remaining manuscripts were transcribed by a professional hired to do so.

### Analysis

Since the aim of the study was to gain an overview of how professionals working in CAP in Sweden describe and frame the phenomenon of TFSV, an interpretative approach such as Reflexive Thematic Analysis is well-suited for the analysis (Braun and Clarke, 2022). Reflexive Thematic Analysis is a theoretically flexible method that facilitates the generation and interpretation of themes from the data, using the researchers’ theoretical viewpoint. We worked inductively at the beginning and did not have a theory-driven approach to constructing the themes; however, we tried to remain open to the material. The process then became more abductive as we read and re-read the material with the codes and themes in mind.

Our ontological positioning is critical realism, which acknowledges a reality that exists beyond language and discourse, while also being aware of the constructive properties of language use. This means that, in the analysis, we viewed the participants’ accounts as constructions of meaning in a contingent space of language; at the same time, we understood these constructions as also being shaped by the material institutional realities in which the participants worked. In the analysis, we drew from a post-structural policy analysis approach, working backward from the proposed solutions and explanations of the problem of TFSV, to understand which assumptions and logics that guide professionals in their conceptualizations of patients and their problems (Bacchi and Goodwin, 2016).

The first step in the analysis was to become familiar with the interview material through reading and re-reading, as well as taking notes on ideas and frameworks that could be used further on in the analysis. The next step was coding the material, which was done in the software Open Code. In parallel with the coding process, notes were taken on possible themes in the material. The final steps, including creating, reviewing, and defining themes, were conducted through discussions among contributing researchers during the writing-up process. In our work with the themes, we alternated between the thematization and the interview material to ensure that the analysis remained grounded in the data.

### Researcher positioning

The principal author, FCR, works clinically in CAP and has met patients exposed to TFSV. Her research interest lies within critical perspectives on psychiatric diagnosis, as well as violence epidemiology and gender. KGG is a researcher within the field of young girls exposed to sexual harassment through technology, mainly in school settings. She has a clear feminist focus in her research. LJ is a researcher working focused on technology-facilitated sexual abuse and IRL child sexual abuse. She is interested in clinical and organizational perspectives on TFSV and how to address this issue within different organizations. The last author, ID, is a researcher and lecturer with a focus on trauma therapy and also works clinically with trauma patients. She has written a book about power issues in psychotherapy processes (Dennhag, 2017).

## Results

In this section, the themes of the interview material will be presented, along with excerpts from the interview that serve to illustrate these themes. The themes reflect the ways professionals made sense of the phenomenon of TFSV in relation to their work with their young patients. The analysis presented in this article adopts a meaning-making perspective. This means shifting the focus away from” objective” facts or experiences that participants contribute and instead highlighting how the professionals discussed and made sense of the phenomenon under study.

The participants’ accounts should therefore be seen as expressions of how CAP understands and frames TFSV, as well as how this phenomenon is positioned about CAP’s role and responsibilities. Although the professionals’ accounts reflect an individualizing framing of TFSV, our analysis seeks to critically unpack this framing and its implications.

The overarching theme of the analysis is *Violence as an individual concern*. An individualization of the issue at hand seems to lay the foundation for how the professionals theorize about how children and young people come to be subjected to TFSV, why this type of violence is harmful, as well as which solutions should be applied within the context of CAP.

Under the umbrella of the individual viewpoint on the phenomenon of TFSV, three main themes were constructed. These three themes illustrate the why (explanations), how (harm dynamics), and what (solutions), in the professionals’ views on the matter. That is, *why* the patients they met were exposed to this type of violence, *what* the harm dynamics of TFSV are specifically, and *how* the professionals imagine that they should handle the harms done (Table 1).

**Table 1:** Findings of the reflexive thematic analysis of interviews with professionals in child and adolescent psychiatry.

Explanations	Violence as an individual concern	
	Harm dynamics	Solutions
It could happen to anyone	The shame and guilt of complying	Releasing shame and guilt
Psychiatric diagnosis	No free zone—the vastness of online space	Establishing right and wrong
Lack of self-worth	Becoming traumatized	Employing strategies to protect
Longing for connection		Processing the trauma
A natural curiosity		

### Explanations (who and why)

#### It could happen to anyone

The first central theme illustrates the professionals’ theories and explanations regarding why the victims of TFSV became subjected to these events. There was a contradictory tension between how the respondents described the characteristics of the victims and their reluctance to make any claims about it. Being exposed to TFSV was, on the one hand, framed as something that could happen to anyone, suggesting it would be evenly distributed among the population of young people. As one of the respondents put it, “*My feeling is that all children could be exposed to this, I mean that there is no specific group of people, but all with different upbringings and different life conditions.” (Participant No. 18).*

On the other hand, the respondents mentioned several grouping characteristics of the victims. First of all, all of the respondents stated that all, or nearly all, of the victims were girls.

*Most of them are, or not most of them, actually all of them which I have treated, were girls. I do not know if that is because it’s more ok to seek help, or if boys just are not exposed to the same extent. I actually do not know. But I have only met girls. (Participant No. 10).*

Another characteristic that almost all respondents mentioned was some kind of neuropsychiatric disorders on the part of the victim: “O*ne thing that I’ve noticed, that I think is common, maybe I’m wrong, but relatively many have neuropsychiatric disabilities (…)” (Participant No. 18)*. A majority of the respondents also stated that in the case of the victims they met, TFSV was seldom the only form of violence exposure:

*I mean, those I’ve met were really in bad condition, but then I have to say that it has not been the only…I mean often there are other types of exposures as well, earlier or later or simultaneously, to violence and abuse. (Participant No. 16).*

Despite the explicit view that this violence “could happen to anyone,” the respondents seemed to have some observations about what characterized these victims.

#### Reaching out for connection

Apart from the demographic characteristics of the victims, the respondents described several psychologically based explanations for why their patients were exposed to TFSV. The respondents perceived that many of the victims they had met had been lonely and in need of acknowledgement and close relationships. “*I think that often they have had a lot of difficulties during their upbringing. Some kind of attachment difficulties. A loneliness. That school never really worked out.” (Participant No. 3).*

The patients were described as lacking support or social competence, or both, and therefore becoming lonely and desperate for attention and recognition. The patients often spent hours at the computer or on their phone, actively searching for some kind of connection with people online.

*Yeah, I mean there was a lot of bullying, since she was very little, kind of ostracized. Had a real difficulty in making relationships. She did not know how to behave, or how to… And then she started seeking attention online. Sending images and getting acknowledgement and validation. (Participant No. 4).*

#### Psychiatric diagnosis

All of the respondents described how neuropsychiatric disabilities played an essential part in how the victims became exposed to TFSV. “*I think that it has been pretty common that these young people have NPF (neuropsychiatric functional* var*iations). That they have a lack of impulse control and awareness of consequences. And an emotional instability.” (Participant No. 3).*

ADHD seemed to be conceptualized as an explanatory factor, where the ADHD-specific difficulties with impulse control and a tendency for sensation-seeking were causing behaviors that heightened the risk for exposure to sexual violence online.

*And I do not know if it’s like the ADHD in itself, or if it is the ADHD that makes it hard for them to have relationships. Which makes them feel lonely, and searching online, and then they are, well, easy prey in some sense. (Participant No. 1).*

#### Lack of self-worth

One factor that was seen as driving risky behaviors and exposing the victims to situations where abuse could happen was a lack of self-worth. This could, for example, be due to earlier abuse, which in the respondents’ narratives could be a factor lowering the feeling of worthiness and self-love.

*I mean the girl had been assaulted IRL. Raped. This was before all of this happened. So maybe that also was part of it. That maybe, or probably, she does not believe that she has a very high worth. (Participant No. 6).*

Some respondents also had met girls with an active desire to hurt themselves, and the respondents were using the concept of sex as self-injury as an explanatory factor.

*And then I think, there is some subgroup that I feel is using sex as a mode of self-injury, even online, which of course does not mean that they do not get traumatized by is, or that is not illegal. But also, that there is some kind of drive in the whole thing. (Participant No. 13).*

#### A natural curiosity

Some of the respondents mentioned that in the beginning of the victims’ contact with the abuser, the contact was often consensual and mutual, and usually based on some kind of real relational and sexual longing and exploration.

*And in all the vulnerability that the victim I situated in, I feel that what is happening is so complex. It’s not only scary and weird and disgusting, but it can also hold a stroke of curiosity, excitement, lust, dependency, joy and belonging. (Participant No. 8).*

Gradually, the situation for the victim could change almost imperceptibly into something other than what it was at the beginning. “*It becomes very unclear for the child, when was it something thrilling and fun and when did it slide over to something I did not want, being scary, weird, frightening.” (Participant No. 8).*

Some of the respondents also described how their patients viewed their interactions, often involving illegal behavior on the part of the other person, as something they gained from and that wasn’t especially problematic. “*In the beginning, when she started to tell me, it was like it was a fun thing to do. Like, do you know what I do, to get some extra cash?” (Participant No. 6).*

### Harm dynamics

#### No free zone: the vastness of online space

The respondents expressed that technology-facilitated abuse had special features, with one feature being the fact that the victims rarely could enjoy free zones from the abuse.

The online-based life of the young people they met also brought forth a new arena of temporal and spatial unboundedness. “*It’s like it takes over their life in a sense. You are never safe. I mean if you are abused in the home, the school might be a safe place. But when it’s on the phone, that you carry all the time. (Participant No. 10).*

The places where the young person could feel safe were thus decimated, and the phone became a mediator of constant abuse.

The temporal and spatial unboundedness of the online space also came with other problems. The respondents described how the images produced of the abuse that happened could resurface on new platforms, a long time after the abuse had stopped, which was often detrimental to the process of healing.

*What I think has been the most tangible part is when you know, or believe, that there are images or films that you (the victim) do not know where they are, or if they might be spread around. I think that is a special feature of these things that makes you less protected. It’s not over, but an ongoing exposure. (…) That you know that the images are in the hands of others, not only the perpetrator. (Participant No. 13).*

This made it hard for the therapists to help the young people feel safe again due to the threat of social shaming should the images get into the wrong hands. “*And I think a lot of shame also, in relation to their friends, feeling scared about where these images are, and who knows about what has happened.” (Participant No. 9).*

#### The shame and guilt of complying

One factor that was framed as an essential driver of distress among the patients was that they often had complied with actions that they could have avoided by just leaving the computer or not responding to the perpetrator. They were not physically forced or threatened, but instead manipulated or verbally coerced into these situations.

*The thing with online abuse is that the young person, or the child, feel that they have been active in a sense. That they, from the beginning, thought that it was a flirt or love relations, or that they were interested or active in the interaction. I have had patients who have been groomed online, and went to see a perpetrator, at several occasions. So, you know, blaming themselves, and thinking about how other people think about it, it’s often a big part. (Participant No. 11).*

The activity on the part of the child was seen as a complicating factor for the alleviation of distress in the aftermath of these kinds of abuse situations.

*Yeah, as I said, many abuse situations that starts with online abuse with someone previously unknown to the child, where the child is an actor and has been active, answering and kind of made the abuse possible, there can be a specific guilt and shame component (…) which might not be there if you were exposed to a situation where you were less active, less an agent. (Participant No. 7).*

#### Becoming traumatized

Most of the respondents described the harm from being exposed to TFSV using the framework of Post Traumatic Stress Disorder (PTSD) or trauma.

*Well yeah, trauma symptoms that have been of PTSD-like character, with different levels within that cluster, where avoidance of course is very strong. Not wanting to think about it or talk about it, not wanting to have anything to do with it, reacting very strongly to intrusive images of memory. (Participant No. 19).*

Within the framework of trauma, the patients’ presenting problems, even self-loathing and doubt, were framed as symptoms of an underlying process of traumatization. This traumatization is, in line with other psychiatric diagnoses, made into a sort of entity in itself, being responsible for the manifesting problems of the patient.

*All trauma related [symptoms]. A lot of anxiety, low mood, a lot of self-loathing, doubt, a lot, yeah. And regulation difficulties, problems with mood, hard to concentrate, social difficulties. Not everyone fits into PTSD (Post Traumatic Stress Disorder), but there are a lot of other signs of traumatization that also are troublesome. (Participant No. 8)*.

### Solutions

#### Releasing shame and guilt

The professionals discussed the weight of shame and guilt that plagued their young patients. This was one of the most important targets for intervention and an integral part of their therapeutic work with the patients they met. “*That it’s not their fault. Grown-ups should not ask for sex from children. That is really not their fault. End of story. It’s about lifting of the shame and the guilt from them in some way.” (Participant No. 3).*

Due to the nature of the crime, the victims’ perceptions of enabling the victimization in a more concrete way were important for professionals to be aware of, and something that could make the release of shame and guilt much harder.

One important thing that the participants wanted to communicate about the aftermath of TFSV was: *That you can be affected by guilt and shame that can be really difficult to cope with yourself, and the guilt is not yours. It all on the offender. (Participant No. 5).*

#### Establishing right and wrong

The respondents emphasized the importance of information as key for both professionals and patients. Becoming informed about how internet-related sexual abuse works, the modus operandi of these victimizations, and on what platforms they happen, was framed as lacking among many professionals, and an important target for intervention to enhance professionals’ ability to do a good job in these cases. “*I think that people with special knowledge about this, its really important to reach out with that knowledge. That you need to understand the phenomenon, that this is how it looks, this is what we know.” (Participant No. 5).*

The respondents also talked about informing patients. This was thought to lessen the bad feelings about what happened through correcting “cognitive distortions” through psychoeducation. The logic behind psychoeducation seemed to be that when informing patients about their issues, it leads to less negative feelings about what happened.

*And it is also a way of working with this, to separate, that there is a distinction between what the child thinks is the truth, and what actually is the truth. In relation to what abuse is. That when you, ok it’s like that, you can start to see things from another perspective (…) So I think the psychoeducational part is very important. (Participant No. 9).*

The professionals further conveyed that many of their patients needed clear messages about whether certain behaviors from other people were ok or not. Legal ramifications were sometimes used as a starting point in discussing such matters with their patients. Information from law enforcement authorities, such as the police, could also be used to make clear the patient’s status as a victim of violence. Since being in Sweden, the law of consent regarding sexual interactions has been a useful tool.

*I think that it’s great that you now can talk about the law of consent. We usually talk about that this law did not exist when I was young, but now it’s there. (…) Sometimes you might have to help a lot, by for example say: you know, now when you have told me it does not really sound like there was any consent here? (Participant No. 2).*

Sometimes, the professionals perceived that they had to take a leading role and clearly explain the difference between right and wrong, legal and illegal, and establish boundaries that had earlier seemed unclear.

*But it often comes to a point where you have to clarify, I mean psychoeducatively, what the difference between legal and illegal is, and the difference between that and norms or what other people think. (Lindas 3 Participant No. 8).*

#### Strategies to protect

This theme describes some coping strategies that the professionals communicated to their patients. Some were with the aim of lowering the risk of being exposed to sexual abuse online. In this case, the most mentioned strategy was blocking the sender. Reporting to the police or other authorities was also mentioned. “*And then talk a lot about how you block, or how you increase security on the apps so that not everyone can get in touch with you or add you and stuff like that. (Lindas 5 Participant No. 10).*

Another strategy aimed to protect the well-being of the patients in the aftermath of TFSV was to help the patients “unknow” what had happened. The problem of TFSV, such as images that were already posted online, was framed as impossible to tackle, and the best way to keep sane was probably to accept that this was going on and that you could not control it.

*Yeah I mean you try to do what you can. I usually recommend to not, if you know that your material is spreading, to try to ‘unknow’ so to speak. To try to not follow, not control, not keep track of this. Because there is nothing you can do anyway. (Participant No. 13).*

#### Processing the trauma

One important solution to patients’ problems in the aftermath of TFSV was to discuss their experiences with a professional.

*Well, that it is good to talk about your experiences, and that I can help to talk about it. That you can begin to feel better. That you can suffer from guilt and shame that can be really difficult to handle on your own. (Participant No. 5).*

Almost all respondents had received training in some form of trauma-focused method. The most common forms were trauma-focused cognitive behavioral therapy (TF-CBT), eye movement desensitization and reprocessing (EMDR) therapy, and prolonged exposure (PE). All of these therapy formats entail some kind of exposure or processing of traumatic experiences.

*I would say that many patients initially (in the treatment) actually feel a bit worse, cause they have tried to push something away for a long time, and when you come here you are not supposed to do that anymore. It’s like cleaning a wound. It hurts, it stings. But when you have a working alliance and when they have gotten that psychoeducation, that it’s not only me this is happening to, it happens to a lot of people. That soothes a little. And then, during the exposure part, many get anxiety, but then it gets better. It goes up and down. But less down the longer into treatment you get. (Participant No. 15).*

The processing of the trauma is described as analogous to cleaning an infected wound. Without cleaning, healing cannot happen. This establishes the professional handling of the trauma problem as essential for the healing and recovery of the patient.

## Discussion

This section expands on the previously presented themes, analyzing them through relevant theoretical concepts and perspectives.

Before presenting the analysis, it is essential to highlight a few points regarding the professionals in the study, as well as emphasize the analytical focus of the article. First of all, the professionals participating in this study were well-versed in the state of the field and updated on how technology worked in aiding in abuse directed toward their patients. Their accounts resonated with much of the empirical research conducted in the area, and they were thoughtful and reflective in their responses. Furthermore, the study was part of a larger investigation aimed at understanding how psychiatric staff address these issues, which prompt responses from a clinical psychiatric perspective and may not allow for consideration of other viewpoints.

The final point to emphasize is that the analysis presented in this article adopts a meaning-making perspective. This means that the focus of analysis is not the facts or experiences that the participants bring with them, but their ways of talking and making sense of the study phenomenon. The accounts are thus viewed as representing how CAP makes sense of TFSV, and how they frame the phenomenon of the task of CAP. The critique or deconstruction applied to these narratives should be viewed in light of this.

The accounts from the professionals bore a high degree of resemblance to one another, suggesting a consensus among the professionals in their view of TFSV. Consensus, on the one hand, can be a sign of strong scientific evidence; however, it can also be a sign of hegemonic discourses, which are defined as values and ideas put forth by people in power that become the dominant way of thinking within a field (Hoffman and Ford, 2010). This is an important distinction given that hegemonic discourses within a professional field could dampen critical discussions and creativity that could enable innovative solutions to the problems faced within the field. Why some conceptualizations become hegemonic within organizations can be connected to larger societal discourses, which we will elaborate on later in the article.

Working backward from the proposed explanations for and solutions to the problem of TFSV about CAP was almost entirely framed as an individual concern. The explanations for why the patients were exposed to TFSV were positioned at the individual level, with intrapsychological as well as neuropsychological driving forces at the core. The harm that comes from exposure to violence was also framed as individual problems and concerns, with a focus on mental distress and symptoms. Regarding strategies for addressing this type of violence from a psychiatric clinical perspective, information transmission as well as processing of internal trauma memories were framed as key factors.

The overarching theme of the analysis, the individualization of violence, is closely tied to larger societal processes of individualization and self-governance. Against the backdrop of the increasing psychologization of human life, increasing problems that were previously deemed social or moral are being addressed by the psy-sciences (Füredi, 2004).

The primary concept used in this article to frame the findings is the phenomenon of psychiatrization, described by Beeker et al. (2021). Psychiatrization is the societal process that stretches the reach of psychiatry, both material and ideological, expanding the notion of what is considered a problem that should be handled within psychiatry and labeling increasing societal problems as mental health disorders. Using a psychiatric lens, problems become symptoms, and symptoms reside within the individual. Psychiatrization is not only thought to be a top–down process, but there is also a bottom–up demand to have one’s problems legitimized and tended to within the psychiatric institution.

The societal process of psychiatrization is, as briefly mentioned, connected to several other processes. One is the growth of therapy culture described by scholars such as Füredi (2004) and Illouz (2008). Other concepts, such as psychologization and medicalization, conceptualized by Conrad and De Vos, also give a backdrop onto which psychiatrization specifically takes place (Conrad, 2007; De Vos, 2010). Another process intertwined with the psychiatrization process, which lowers the thresholds for unacceptable experiences, is the creep of harm-related concepts described by Haslam (Haslam and McGrath, 2020). These concepts are, in various ways, related to a broader tendency in our time to focus on the individual and emphasize agency, while minimizing or ignoring the importance of societal structures. This focus tends to give precedence to medical or medically framed interventions, which in turn promote individual coping strategies for problems that could be conceptualized as social, rather than encouraging more structural or community-based solutions.

Psychiatrization might not be a problem for individual patients. However, when including an issue such as TFSV in the scope of psychiatry, this could signal to society that the problem is a medical concern and is being addressed by medical institutions. This, perhaps unintentionally, shifts the focus to individual solutions and directs the gaze to the aftermath of violence rather than its prevention. The process of psychiatrization can also mean that psychiatry’s institutional perspectives on an issue gradually influence how the general public understands it. This view further serves to obscure perspectives of inequality, that is, the fact that this type of violence might not be evenly distributed in the population but disproportionately affects young girls and especially girls with previous exposure to violence. Despite research demonstrating the strong social gradients in health, studies have shown that even when policy explicitly acknowledges the role of structural societal problems in the development of poor mental health in the population, the proposed solutions remain at an individual level (Fjellfeldt, 2023; Marmot, 2015).

Regarding the reasons professionals provide for why their patients were exposed to TFSV, the reluctance among the professionals to characterize the victims or draw conclusions about them contrasts with the specific observations they had actually made. Their hesitation toward stereotyping patients could be understood as a therapeutic openness and resistance to being normative, as well as a recognition of the limitations of so-called anecdotal evidence. However, it could also be seen as yet another sign of the individualistic viewpoint echoing throughout the narratives. Two demographically and structurally related observations became salient in how the professionals perceived the characteristics of the TFSV-exposed patients: they almost always were girls, and they more often than not had been subjected to other forms of violence in addition to TFSV. It is not surprising that the majority of the patients were girls, in light of the epidemiology of sexual violence (e.g., Swedish Crime Council, 2014). There might be obstacles for boys exposed to TFSV to reach mental health care, such as a reluctance to disclose exposure or not being asked about sexual violence when expressing mental health concerns. However, there is still a constant overrepresentation of girls and women in the literature when it comes to sexual violence.

The fact that the patients were perceived as multiexposed to violence is also something that resonates well with the empirical epidemiology of violence shown in both trauma and criminology research studies (Curiel et al., 2018; Rayment-McHugh, 2023; Wolfe, 2018). Why these structural inequalities were unproblematized by the participants in the study can be traced back to the individualized subject positions offered through psychiatric language (Brinkmann, 2020).

When elaborating on why their patients had been exposed to TFSV, individual perspective took precedence and elicited several intrapsychological theories explaining why these particular patients became victims. The one explanatory factor that seemed most salient for the professionals and was mentioned as a factor by all the participants was that the patients exposed to TFSV very often had neuropsychiatric disorders, where ADHD was the most mentioned diagnostic label. ADHD was described in explanatory terms, conceptualized as a driving force behind the behavior that led the patients to end up in unsafe situations, heightening their risk of being exposed to TFSV. Reification of psychiatric disorders is a typical cognitive process, both among lay people and professionals, and means that the psychiatric diagnostic label is made into an entity, or latent construct, that can be used in a circular explanation of behaviors and mental states. In the case of our respondents, one of the attention-deficit/hyperactivity disorder (ADHD) core symptoms, namely impulsivity, was used as an explanation for victims’ tendency to find themselves in risky situations. This exemplifies the logic of circular reasoning, since impulsivity is also one of the symptoms that led to the patient receiving the diagnosis in the first place (te Meerman et al., 2022). Why ADHD lends itself so readily to becoming an explanation of why people act the way they do might be understood in light of how the language of psychiatry has become the primary lens through which suffering and dysfunction are viewed (Brinkmann, 2020). Furthermore, diagnostic labeling often aims to serve as a kind of moral unburdening of the patient. Because diagnostic labels imply that an inner malfunctioning module in the brain is responsible for patients’ problems, the blame is not attributed to the patient’s self. For more elaborate discussions of the appeal of brain-based explanations, see (Bennett and McLaughlin, 2024). However, this unburdening is seldom a straightforward process, and research shows that diagnostic labels may not alleviate stigma to the extent hoped for (Seery et al., 2021).

A less neurobiological but more emotionally, or intrapsychologically, related factor that the professionals used as an explanation for the patient’s exposure to TFSV was loneliness. Often, as a result of having difficulties in acquiring and retaining satisfying relationships. The cross-sectional association between loneliness and problematic online behavior, such as excessive online time or seeking relationships with strangers, is well-established in the empirical literature (Domoff et al., 2024). When studied longitudinally, however, victimization is shown to precede the experience of loneliness (Gonggrijp et al., 2023). Loneliness was also one of several intrapsychological factors known to be associated with self-harming behavior, such as using sex as a mode of injury to oneself. The complex behavior that constitutes self-harm was also reified and made into a driving force, causing the patient to seek out dangerous situations that brought about risk for sexual victimization.

Several of the harm dynamics that the participants described as affecting their patients are valid for other types of sexual violence as well, but certain factors are thought to be enhanced or facilitated by the technological aspects. One such thing is the temporal and spatial unboundedness of online communication (Hamilton-Giachritsis et al., 2020). Many young people carry their phones with them constantly, and perpetrators are therefore not limited by time and space when getting access to their victims. Another factor that was described as exacerbated by the vastness of the online space was the ongoing trauma or vulnerability that images of sexual abuse cause the victims. This is described in the literature of IRL sexual violence as well, but has become even more pressing due to the infrastructure of the internet, which enables sharing and distributing images very rapidly and to all corners of the world (Gewirtz-Meydan et al., 2018; Schmidt et al., 2024).

Both of the factors mentioned above connect well to one of the main components of the suffering endured by TFSV victims, as described by the professionals in the present study, namely, the emotion of shame. Shame is a well-established component of the psychological aftermath of sexual violence. It is often mentioned by clinicians as something that can delay disclosure of abuse as well as be detrimental to the mental health of sexual violence survivors. Shame is usually expressed as something undeniable and natural among victims of sexual violence. However, recent affect theories contradict core emotion theories and suggest that emotions are very much culturally embedded (Barrett and Russell, 2015). Shame might thus be something that both victims of sexual violence and their therapists expect to see, and the discourse of it being natural and obvious might also bring it about (Weiss, 2010). Shame often makes the individual want to hide and shield. In contrast, other feelings, such as indignation or a sense of injustice, can lead to more constructive actions that focus on addressing the perpetration of violence as the primary issue.

Within psychiatric and psychological understanding of the concept of trauma, the preferred solution proposed in dealing with the aftermath is some kind of trauma processing. This is something that is done in several forms of trauma therapy (e.g., Foa and Kozak, 1986). The rationale is that the traumatic impact of an adverse event is stuck in the body and/or brain, and the analogy of cleaning a wound is often used as an illustration of what is done when trauma is processed (Cohen et al., 2012). You go in, think about what happened, together with regulatory support from a therapist, which is analogous to scrubbing the dirt out of the wound. Afterward, the wound can fully heal. Many of the issues of the victims of TFSV were conceptualized within the framework of trauma or PTSD. Trauma as a concept is often applied when there is a known event in the biography of the patient that is thought to evoke traumatic responses.

The concept of trauma was hoped to highlight harm earlier unrecognized, as well as counter pathologization of psychiatric symptoms that were caused by traumatic experiences and not innate dysfunction (Wilbur, 1990). Instead of achieving these aims, the concept has in many ways served to obscure the focus on structural inequalities and systemic violence (Chatterjee and Park, 2024). Some scholars have criticized what they name the *trauma of rape* discourse, which states that rape causes an inevitable psychological scarring with life-long implications, and that professional help often is needed to heal. The primary critique is that this discourse serves to disempower women and naturalize specific reactions, thereby stifling political action and the expression of dissent (Gavey and Schmidt, 2011). In summary, each of the conceptualized explanations, harm dynamics, and solutions reflected in the participants’ accounts lacks awareness about structural inequalities that shape the lives of many, or even most, of the patients seen in psychiatric care.

It is clear that through the concept of trauma, violence, subjection, and perpetration can become a concern for psychiatry. When the professionals are framing their patients’ problems in medical terms, such as highlighting traumatic symptoms and neuropsychiatric disorders, they use the terminology of psychiatry to ensure access to support and care for a group that needs it. To understand why this happens, it is essential to consider the structural conditions in which the professionals are situated. Psychiatry is organizationally embedded within the language and structure of medicine and health care, which means that certain practices are encouraged while others are made more or less impossible. There is also an economic structure dictating much of the scope of the practice. All of this makes specific ways of perceiving, working, and framing a problem more probable than others. Through the use of diagnostic labels and a medically framed language, the patients’ problems become legitimized within the institution. Making violence a psychiatric concern is to make it an individual concern. In a sense, the very process that obscures the societal view on the problem is also the one needed to ensure its legitimacy.

## Conclusion

This article analyzed the conceptualizations of TFSV by professionals working in CAP. It contributes a new perspective on the phenomenon of TFSV from the vantage point of CAP as an institution. It also contributes a critical perspective on the psychiatrization of this new phenomenon.

In summary, the findings show that professionals in CAP tend to individualize the issue of TFSV, focusing on individual factors involved in exposure, harm, and interventions.

Further research is needed on how TFSV can be addressed, and societal debate on institutional responses to this problem is encouraged.

## Strengths and limitations

One of the limitations of the present study is the lack of explicit questions investigating the professionals’ views on the phenomenon of TFSV on a structural level. This was due to the overarching aim of the larger study, which was to investigate the approaches to addressing the problems of TFSV in CAP clinics. This may have obscured other perspectives that the professionals had to offer.

Another limitation is the transferability of the findings due to the small sample size and local recruitment. This could be enhanced by future research covering a broader selection of CAP services.

Future research should include other social institutions that respond to the issue of TFSV and contrast their findings and analysis with those presented in this article. Research should also look into detail regarding the diagnostic considerations that clinicians discuss when meeting patients exposed to these “new” traumatic events.

## Data Availability

The datasets presented in this article are not readily available because the correspondent author do not have full ownership of the dataset since it is a part of a larger project. Requests to access the datasets should be directed to frida.rindestig@umu.se.
